# Joint inflammation assessed by physical examination and MRI of the knee in juvenile idiopathic arthritis: low predictive value for synovitis

**DOI:** 10.1186/1546-0096-12-S1-P2

**Published:** 2014-09-17

**Authors:** Charlotte M Nusman, Robert Hemke, Dieneke Schonenberg, Koert Dolman, Marion van Rossum, Merlijn van den Berg, Mario Maas, Taco Kuijpers

**Affiliations:** 1 Radiology, Academic Medical Center, Netherlands; 2 Pediatric Hematology, Immunology, Rheumatology and Infectious Diseases, Emma Children's Hospital, AMC, Netherlands; 3 Pediatric Hematology, Immunology, Rheumatology and Infectious Disease, Emma Children's Hospital AMC, Netherlands; 4 Pediatrics, Sint Lucas Andreas Hospital, Netherlands; 5 Pediatric Rheumatology, Reade Institute, location Jan van Breemen, Netherlands; 6 Pediatrics, Emma Children's Hospital AMC, Amsterdam, Netherlands

## Introduction

The presence of joint inflammation in juvenile idiopathic arthritis (JIA) patients can be made by physical examination and confirmed by imaging. The discrepancy between physical examination and MRI for evaluation of synovitis in a target joint is possibly explained by the fact that clinical measures mostly reflect overall disease activity instead of measures specific for the joint imaged by MRI.

## Objectives

To compare clinical disease activity of the major target joint upon physical examination with a validated MRI score for the knee in JIA.

## Methods

MRI datasets and corresponding clinical parameters of disease activity of the knee were analyzed in 167 JIA patients (61.7% female, mean age 12.8 years, SD 3.4 years). Local physical examination of the knee included absence or presence of swelling, warmth, pain or limitation-of-motion (LOM) as assessed by experienced pediatric rheumatologists. A blinded radiologist (6 years of experience in MRI in JIA) analyzed synovial hypertrophy (SH) on a scale from 0-12 on all MRI datasets following the validated Juvenile Arthritis MRI Scoring system (JAMRIS). SH was ‘present’ when the total JAMRIS score was >2. Diagnostic accuracy of the local physical examination parameters for detection of arthritis was determined with MRI as reference standard.

## Results

Sensitivity and specificity of the parameters scored by local physical examination compared with MRI varied from 39-71%. The overall positive predictive value for synovitis was very low (21-28%), while the negative predictive value was relatively good (71-74%). Median time between the clinical assessment and the MRI was 38 days (IQR 28-53 days). Subgroup analysis on 51 patients with <31 days (median 25 days) between clinical assessment and MRI did not improve the diagnostic accuracy.

## Conclusion

The presence of swelling, warmth, pain or LOM on physical examination did not predict the presence of synovitis upon MRI. The time between clinical assessment and MRI appeared to have no influence on the diagnostic accuracy of the physical examination inflammation parameters. While the discrepancy between physical examination and MRI persists, follow-up studies are warranted to unravel the difficulties in assessment of disease activity.

## Disclosure of interest

None declared.

